# Reciprocal Effects of Silicon Supply and Endophytes on Silicon Accumulation and *Epichloë* Colonization in Grasses

**DOI:** 10.3389/fpls.2020.593198

**Published:** 2020-10-27

**Authors:** Ximena Cibils-Stewart, Jeff R. Powell, Alison Jean Popay, Fernando Alfredo Lattanzi, Sue Elaine Hartley, Scott Nicholas Johnson

**Affiliations:** ^1^Hawkesbury Institute for the Environment, Western Sydney University, Penrith, NSW, Australia; ^2^Instituto Nacional de Investigación Agropecuaria, Colonia, Uruguay; ^3^AgResearch, Ruakura Research Centre, Hamilton, New Zealand; ^4^Department of Animal and Plant Sciences, The University of Sheffield, Sheffield, United Kingdom

**Keywords:** silica, *Epichloë*, hydroponics, perennial ryegrass (*Lolium perenne* L.), tall fescue (*Festuca arundinacea* S.)

## Abstract

Cool season grasses associate asymptomatically with foliar *Epichloë* endophytic fungi in a symbiosis where *Epichloë* spp. protects the plant from a number of biotic and abiotic stresses. Furthermore, many grass species can accumulate large quantities of silicon (Si), which also alleviates a similar range of stresses. While *Epichloë* endophytes may improve uptake of minerals and nutrients, their impact on Si is largely unknown. Likewise, the effect of Si availability on *Epichloë* colonization remains untested. To assess the bidirectional relationship, we grew tall fescue (*Festuca arundinacea*) and perennial ryegrass (*Lolium perenne*) hydroponically with or without Si. Grasses were associated with five different *Epichloë* endophyte strains [tall fescue: AR584 or wild type (WT); perennial ryegrass: AR37, AR1, or WT] or as *Epichloë*-free controls. Reciprocally beneficial effects were observed for tall fescue associations. Specifically, *Epichloë* presence increased Si concentration in the foliage of tall fescue by at least 31%, regardless of endophyte strain. In perennial ryegrass, an increase in foliar Si was observed only for plants associated with the AR37. *Epichloë* promotion of Si was (*i*) independent of responses in plant growth, and (*ii*) positively correlated with endophyte colonization, which lends support to an endophyte effect independent of their impacts on root growth. Moreover, *Epichloë* colonization in tall fescue increased by more than 60% in the presence of silicon; however, this was not observed in perennial ryegrass. The reciprocal benefits of *Epichloë*-endophytes and foliar Si accumulation reported here, especially for tall fescue, might further increase grass tolerance to stress.

## Introduction

Symbiotic relationships between plants and fungi have a long evolutionary history with plant fossils containing fungal endophytes dating back 400-million-years (Krings et al., 2007). Worldwide, many cool-season (C_3_) grasses, including important wild and domesticated species, associate asymptomatically with *Epichloë* fungi (Ascomycota: Clavicipitaceae) (Leuchtmann, 1992; Kauppinen et al., 2016). Asexual *Epichloë* endophytes reside intercellularly (apoplastic space) in aerial plant parts, and are vertically transmitted *via* host seed (Leuchtmann, 1992; Christensen et al., 2008). *Epichloë* endophytes are true obligate symbionts, and their growth is tightly synchronized with their host plant (Christensen et al., 2008). *Epichloë-*grass associations are known to benefit grasses in a number of ways, including increased growth (Gundel et al., 2013), better tolerance to water deficits (Rho et al., 2018) and resistance to pathogens (Xia et al., 2018) and herbivores (Bastias et al., 2017), the latter mainly via production of endophyte specific protective alkaloids (nitrogenous compounds) (Bastias et al., 2017). While some studies suggest endophytes play a limited role in stress alleviation (Niones and Takemoto, 2014; Wiewióra et al., 2015; Heineck et al., 2020), and might have antagonistic responses under extreme resource limitations conditions (Cheplick et al., 1989; Cheplick, 2007; Saikkonen et al., 2016), it is recognized that their beneficial effects are now widely reported (Kuldau and Bacon, 2008; Perez et al., 2017). Because of these benefits, animal-safe endophytes strains (e.g., AR1, AR31, AR584), that don’t have the genes accountable for producing mammalian toxic alkaloids, are commercially available in marketed varieties of several perennial forage grasses such as tall fescue, perennial ryegrass, and cocksfoot (Gundel et al., 2013; Kauppinen et al., 2016). These animal-safe strains improve forage quality and persistence, and maintain endophyte-mediated resistance to insects pests without affecting grazing mammals (Gundel et al., 2013); some negative repercussions have been reported for horses, however (Munday et al., 2017).

Additionally, it is long known that grasses are high accumulators of silicon (Si) (Raven, 1983), especially perennial species that co-evolved with herbivores (Massey et al., 2007; Cooke and Leishman, 2011). Si can account for up to 10% of dry mass, and grasses take up more Si than any other inorganic constituent (Epstein, 1994; Kumar et al., 2017). Silicon is taken up by the roots as monosilicic acid (H_4_SiO_4_), using either passive (transpiration flow) or active (membrane transporters) mechanisms, before polymerizing in cell walls (Ma and Yamaji, 2015; Coskun et al., 2019). Plant silicification (anamorphous Si deposits) occurs in cell walls, cell lumens, intercellularly (apoplastic space), or within leaf surfaces (e.g., trichomes or phytoliths) (Kumar et al., 2017). Once polymerized, Si cannot be remobilized (Hartley et al., 2015). Silicification of plant tissues has been shown to alleviate a wide range of stresses, some of which are similarly alleviated by *Epichloë* endophyte associations. Stresses alleviated by Si include herbivory (Hartley et al., 2015; Reynolds et al., 2016), pathogens (Vivancos et al., 2015; Rasoolizadeh et al., 2018), low temperatures (Zhang et al., 2008), UV radiation (Tripathi et al., 2017), and nutrient deficiency (Miao et al., 2010). The mechanisms underpinning this stress alleviation remain controversial but silicification of plant tissues may provide physical resistance (Coskun et al., 2019) or indirectly cause changes in plant chemistry (Hall et al., 2019). Moreover, Si supplementation has also been reported to increase plant growth (Frew et al., 2018) and enhance uptake of major essential nutrients (Eneji et al., 2008), although these effects mostly take place during stressful conditions (Coskun et al., 2019). For these reasons, Si is promoted as a sustainable fertilizer (Eneji et al., 2008).

Despite endophytes and Si accumulation being important for many grass species, and given that they perform similar functions, their effects have mostly been studied separately. Thus, it is currently unknown if and how *Epichloë* endophytes and Si interact (Johnson et al., 2016). To date, only one field study noted that meadow fescue (*Festuca pratensis*) colonized with *Epichloë uncinata* contained 16% more Si relative to non-symbiotic plants (Huitu et al., 2014). Although this field study did not control for Si-availability, it provides indirect evidence that that endophytes might increase Si concentrations in grasses.

Several putative mechanisms could be envisaged for endophytes and Si impacting one another. For instance, both endophyte (Christensen et al., 2008) and Si (Kumar et al., 2017) can occupy the same apoplastic (intercellular) space in leaves. Therefore, Si deposition could reduce spatial niches for *Epichloë*-mycelium, thereby hindering colonization. Additionally, both endophyte (Cheplick and Faeth, 2009) and Si (Ma and Yamaji, 2015) acquisition come at a metabolic cost to the plant, thus, there may be an optimal balance between Si accumulation and endophyte colonization. Finally, although *Epichloë* colonize aboveground plant tissues, they are known to influence several belowground plant functions, including nutrient/mineral acquisition, thus, may increase Si uptake. These effects are rather indirect, mediated by changes in plant growth. For instance, *Epichloë* can increase leaf area and transpiration rate, and thus nutrient acquisition via mass flow (Malinowski and Belesky, 2000; Soto-Barajas et al., 2016; Llorens et al., 2019), can promote changes in root growth and architecture (e.g., increasing length, reduced diameter, and longer hairs) that increase nutrient concentration gradients (Malinowski and Belesky, 2000; Ren et al., 2007; García Parisi et al., 2015) or facilitate interception with nutrient pools in the soil (Malinowski et al., 1998, 2004; Malinowski and Belesky, 2000; Ren et al., 2007; Soto-Barajas et al., 2016). Endophytes might affect soil organisms that actively facilitate nutrient absorption processes (e.g., arbuscular mycorrhizae fungi) (Novas et al., 2011). Likewise, endophyte can increase root exudation of phenolic compounds that acidify the rhizosphere and improve mineral uptake (Malinowski et al., 1998, 2004).

The objective of this study was to determine whether Si interacts with different *Epichloë* endophytes strains in the two most used perennial pasture grasses worldwide; tall fescue and perennial ryegrass. Specifically, five different *Epichloë* strains were utilized, three animal-safe strains, AR584 in tall fescue, and AR1/AR37 in perennial ryegrass, as well as their wild type (WT) (mammalian toxic) counterparts for both grasses; along with *Epichloë*-free controls. Both extensively used commercial animal-safe (novel) and WT strains were utilized to determine if strain-specific effects occur. In particular, we addressed three specific research questions:

(i) Do *Epichloë*-endophytes increase Si concentrations in foliage?
(ii) Is endophyte-mediated variation in Si concentration dependent of symbiosis induced changes in plant growth?
(iii) Does Si supplementation affect endophyte colonization?

## Materials and Methods

### Experimental Procedure

Seeds of tall fescue (*Festuca arundinacea)* cv. INIA Fortuna and perennial ryegrass (*Lolium perenne*) cv. Samson were obtained from the Margot Forde Germplasm Centre (Palmerston North, New Zealand). *Epichloë* strains utilized were the novel AR584 and the common-toxic (WT) in tall fescue, and the novel AR1 and AR37, and the common-toxic (WT) for perennial ryegrass, along with *Epichloë*-free controls (Nil) (Hume et al., 2013) (see section “*Epichloë* Detection and Mycelium Mass”). These germplasm was utilized to evaluate the effect of endophyte on foliar silicon concentrations.

All seeds were surface sterilized and transferred to trays with wet perlite growing media in a naturally lit glasshouse under controlled environmental conditions of 22/18°C (day/night), 50% relative humidity, at the Hawkesbury Institute for the Environment, Richmond, NSW, Australia. Eleven-days after germination, uniformly sized individual seedlings were transferred to polypropylene 50-mL LightSafe tubes (Sigma-Aldrich, St. Louis, MO, United States) containing 45-mL of nutrient solution with (+Si) or without (−Si) silicon (Si), as described in Hall et al. (2020) adapted from Jung et al. (2015) and Hall et al. (2020).

The solution contained: 1 mM KNO_3_, 1 mM Ca(NO_3_)_2_, 1 mM KH_2_PO_4_, 0.6 mM MgSO_4_, 100 μM NaCl, 15 μM H_3_BO_3_, 0.5 μM MnCl_2_, 0.7 μM ZnSO_4_, 0.8 μM Na_2_MoO_4_, 0.8 μM CuSO_4_, 100 μM NaFe EDTA (Sigma Aldrich, St. Louis, MO, United States). To generate +Si treatments, liquid potassium silicate was added to the nutrient solution (K_2_SiO_3_; Agsil32, PQ Australia, SA, Australia) at a concentration equivalent to 2 mM SiO_2_. Chemically, silicic acid polymerizes to form silica gel (SiO_2_ nH_2_O) when the concentration of silicic acid exceeds 2 mM (Ma and Yamaji, 2006). To balance the additional potassium (K) and chloride (Cl) ions in the +Si treatments, potassium chloride (KCl) was added to the control nutrient solution (−Si). Lastly, the pH of both solutions (+Si/−Si) was adjusted to 5.6-6 using hydrochloric acid (2M HCl) to reduce the polymerization of silicates (Ma and Yamaji, 2006).

The combination of grass species, endophyte strain and Si supply resulted in 14 treatments performed in two experimental stages. Experimental stages were deployed 4 weeks apart with at least 10 replicates of each treatment (183 plants in total; see %Supplementary Table S1 for initial and final replication). The first stage was limited to tall fescue either infected with AR584 or not (Nil) and supplied with (+Si) or without Si (−Si), resulting in four treatments. The second stage included all *Epichloë* by genotype combinations tested in a factorial combination with and without Si.

Plants were grown in their corresponding Si treatment for a further 7 week period to ensure functional *Epichloë*-grass symbiosis (Hume et al., 2013; Kaur et al., 2015). Tubes within the glasshouse were shifted randomly every week to minimize position bias, and the nutrient solution was refreshed three times a week to ensure optimal growth conditions.

At each harvest (Supplementary Figure S1) two of the thickest tillers of each plant were blotted for *Epichloë* detection (see section “*Epichloë* Detection and Mycelium Mass”). Individual shoots and roots were separated, oven-dried at 60°C, weighed (MS-TA Analytical balances; Mettler Toledo) and milled.

### *Epichloë* Detection and Mycelium Mass

Before initiating experiments, 100 mature seeds from each germplasm were stained with aniline blue and examined under the microscope (40×) to corroborate presence or absence (Nil) of endophyte hyphae in the aleurone cells (Latch and Christensen, 1985). Histological detection confirmed infection rates higher than 95% in infected seeds, and less than 3% infection in Nil. However, seed detection does not test endophyte viability.

**FIGURE 1:**
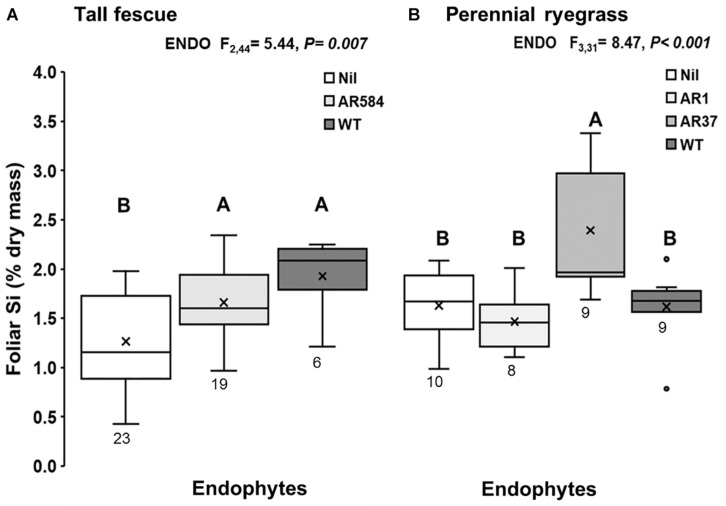
Foliar Si concentration of **(A)** tall fescue and **(B)** perennial ryegrass plants without endophytes (Nil) or colonized with different *Epichloë* endophyte strains growing hydroponically in the presence of silicon (Si). Mean values indicated with ‘X’ and lines depict inclusive median and interquartile range; resulting N indicated below each box plot (see %Supplementary Table S1). Different letters indicate significant differences (*P* < 0.05).

**FIGURE 2:**
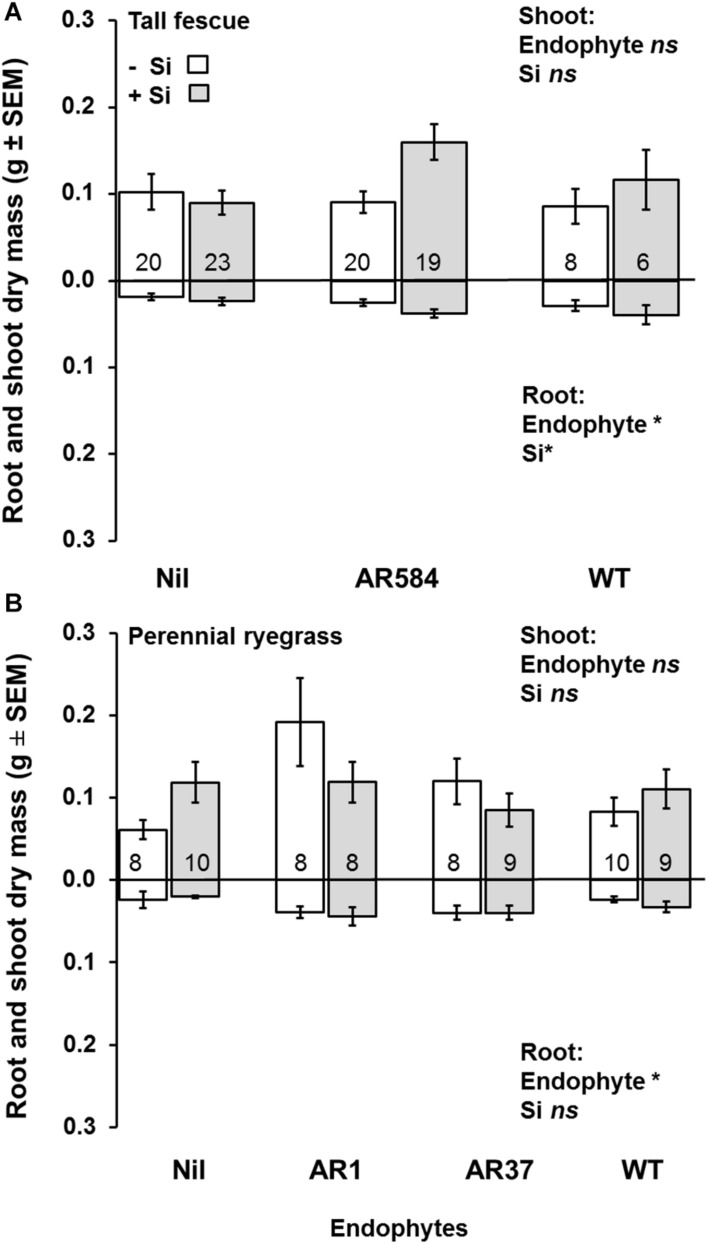
Variation in shoot (top) and root (bottom) dry mass for **(A)** tall fescue and **(B)** perennial ryegrass plants without endophytes (Nil) or colonized with different *Epichloë* endophyte strains growing hydroponically in the absence (−Si) or presence (+Si) of silicon (Si). Mean values ± standard error shown with N indicated within each bar. N is unbalanced due to removal of plants with wrong endophyte status (see %Supplementary Table S1). Models with significant main effects and/or interactions (*P* < 0.05) noted with *, ns = non-significant effects (see Table 1 for full analysis).

Consequently, *Epichloë* viability *in planta*, a measure of vegetative colonization efficacy, was performed in all 183 experimental plants using tissue-print immunoblotting and histological detection immediately after harvest (Hahn et al., 2003). For tissue-print immunoblotting, the fresh cut end of each tiller was pressed onto a nitrocellulose membrane (Hahn et al., 2003; di Menna et al., 2012). Tissue-print immunoblotting results were further confirmed through histological staining (aniline blue), whereby a section of the epidermal strips from the outermost leaf sheath of 20% of the plants in each *Epichloë* germplasm combination (*n* = 7) were examined under a light microscope to corroborate presence or absence (Nil) of endophyte hyphae in intercellular spaces (di Menna et al., 2012). Immunoblotting results and histological tissue staining coincided for the subset of plants evaluated. A total of 17 plants had the wrong endophyte status (e.g., Nil that were positive) and were removed from all analyses (final replication provided in %Supplementary Table S1 and in Figures 1, 2, 4).

*Epichloë*-mycelial mass was further quantified in at least five replicates per treatment (%Supplementary Table S2) using sandwich ELISA following Easton et al. (2002) procedures, modified from Miles et al. (1998). *Epichloë*-mycelial mass was used as a measure of tissue-colonization (Easton et al., 2002). Briefly, approximately 20 mg of milled shoot tissue per plant were utilized. Samples were extracted utilizing 10 mL of phosphate buffered saline with Tween (1%), inverted to mix, incubated with the anti-endophyte antibody for 3 h at 37°C, and stored in the fridge overnight (4°C). An aliquot of 150 μL was taken for the ELISA. ELISA standard curves were prepared for each assay using *Neotyphodium lolii* (now *E. festucae* var. *lolii*) as standards (Miles et al., 1998; Easton et al., 2002). To minimize the effects of interplate variation, each grass sample was analyzed twice. A_405_ was measured with a Bio-Rad model 3550 microplate reader. The plates were washed three times with PBS after the antibody-coating and -blocking steps and twice with PBS-Tween (0.05%) and once with PBS after the sample and conjugate incubation steps (Miles et al., 1998; Easton et al., 2002). Undetectable levels of mycelium following sandwich ELISA confirmed absence of endophyte in all Nil plants.

### Foliar Si

Foliar Si concentration was measured on all replicates (166 plants) on ∼80–100 mg of ground shoot tissue using X-ray fluorescence spectrometry (Epsilon 3x; PANalytical, EA Almelo, Netherlands), following Reidinger et al. (2012) procedures.

Specifically, milled samples were placed individually inside small-mass holders (PANalytical, B.V., Netherlands), and Si concentrations were measured in the presence of helium (Hiltpold et al., 2016). Si was expressed as % of dry mass, and samples were calibrated against a certified control (NCS ZC73018, Citrus leaves, China National Institute for Iron and Steel) (Hiltpold et al., 2016). −Si treatment was verified, given that all −Si plants contained undetectable levels of Si (Hiltpold et al., 2016).

### Statistical Analysis

R (version 4.0.0; R Core Team, 2020) was utilized for all statistical analyses. Sample sizes were unbalanced due to removal of plants with wrong *Epichloë* status (%Supplementary Table S1). Assumptions of normality for residuals were verified according to inspection of quantile–quantile plots. Moreover, since the experiment was performed in two time-lapsed stages, experimental ‘stage’ was accounted as a random effect in all models (%Supplementary Table S1).

Root, shoot, and total biomass were analyzed with a two-way ANOVA with endophyte and Si status, and their interaction included as factors for each grass species in separate models. Biomass traits were additionally analyzed using a one-way ANOVA for each endophyte strain individually with Si status as a factor. Since endophytes increased root growth (see below), we tested the independent effects of endophytes on foliar Si concentration using a one-way ANCOVA, with endophyte status as a fixed factor and root mass fitted as a covariate. Additionally, a Spearman’s rank correlation coefficient test using the *‘cor.test’* function in R was commuted to analyze the relationship between foliar Si concentrations and *Epichloë* colonization for each *Epichloë*-strain separately. Lastly, colonization of each *Epichloë*-strain was analyzed with a one-way ANOVA using Si treatment as a predictor to determine the effects of Si on endophyte mycelium mass (proxy for colonization).

## Results

Results showed evidence of strong interaction between *Epichloë* endophyte and Si. The presence of *Epichloë* consistently increased foliar Si concentrations in tall fescue plants with increases of 31% in plants associated with AR584 and 52% in plants associated with WT endophyte, relative to *Epichloë*-free (Nil) plants (Figure 1A). In perennial ryegrass, foliar Si concentrations increased by 47% only in plants associated with AR37 endophyte (Figure 1B); while the other plants displayed similar concentrations to those of Nil plants (Figure 1B).

Neither endophytes nor the addition of Si influenced shoot biomass for either grass species. Root biomass, however, was significantly increased by endophytes in both species (Figure 2 and Table 1). Including root dry mass as a covariate in ANCOVA indicated that the increased root mass associated with endophyte presence did not fully explain observed increases in foliar Si concentrations in either grass species; tall fescue (*F*_1_,_44_ = 1.55, *P* = 0.22*)* nor perennial ryegrass (*F*_1_,_31_ = 1.63, *P* = 0.21). In short, foliar Si concentrations were higher in endophyte colonized plants irrespective of the increased root mass observed in these plants. When traits were analyzed for each endophyte strain separately, Si significantly increased shoot, root, and total biomass only for AR584-symbiotic tall fescue (Table 2). Other than for AR584, Si did not impact growth traits of the other strains tested in either tall fescue or in perennial ryegrass (Table 2).

**TABLE 1:** Results from multiple comparison tests (Tukey-adjusted *P*-values) for changes in plant traits as affected by Si and *Epichloë* endophytes for tall grass fescue and perennial ryegrass.

			Shoot dry mass		Root dry mass		Total dry mass	
Grass species	Factors	*df*	*F*	*P*	*F*	*P*	*F*	*P*
Tall fescue	Si	*1,90*	3.02	0.085	5.14	**0.026**	3.39	0.051
	Endophyte	*2,90*	1.61	0.204	3.93	**0.023**	2.06	0.133
	Si × Endophyte	*2,90*	2.92	0.058	0.36	0.069	2.55	0.083
Perennial ryegrass	Si	*1,62*	0.03	0.871	0.26	0.611	0.001	0.984
	Endophyte	*3,62*	2.25	0.090	3.25	**0.027**	2.823	**0.045**
	Si × Endophyte	*3,62*	2.33	0.082	0.29	0.834	1.782	0.159

*Models with significant (P < 0.05) main effects and/or interactions are noted in bold.*

**TABLE 2:** Results from the multiple comparison tests (Tukey-adjusted *P*-values) for changes in plant traits as affected by Si on individual *Epichloë* endophyte strain for fescue and ryegrass, respectively.

				Shoot dry mass		Root dry mass		Total dry mass	
Grass species	Strain	Factor	*df*	*F*	*P*	*F*	*P*	*F*	*P*
Tall fescue	Nil	Si	*1,41*	0.256	0.615	0.81	0.371	0.06	0.117
	AR584		*1,37*	8.44	*0.006*	4.33	***0.044***	8.81	***0.005***
	WT		*1,12*	0.66	*0.430*	0.81	*0.386*	0.74	*0.406*
Perennial ryegrass	Nil	Si	*1,16*	3.75	0.071	0.14	0.070	2.74	0.117
	AR1		*1,14*	1.53	*0.230*	0.13	*0.716*	1.14	*0.304*
	AR37		*1,15*	1.10	*0.310*	0.01	*0.998*	1.00	*0.410*
	WT		*1, 17*	0.91	*0.353*	1.52	*0.234*	1.24	*0.280*

*Models with significant main effects (P < 0.05) are noted in bold.*

**FIGURE 3:**
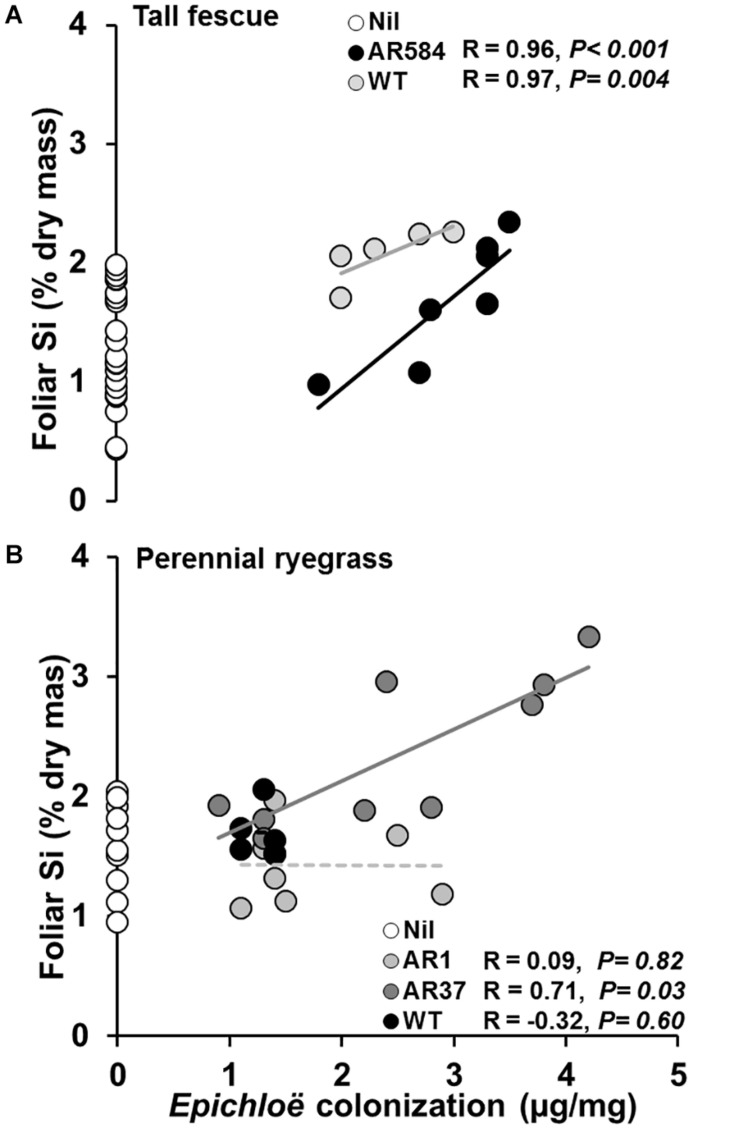
Relationships between *Epichloë* endophyte colonization and foliar Si concentration in **(A)** tall fescue and **(B)** perennial ryegrass with (filled circles) and without (Nil, white circles) endophytes growing hydroponically in the presence of silicon (Si). Lines represent linear regression through data points for each endophyte strain tested. Significant and non-significant relationships represented as solid or dashed lines, respectively. White circles represent Si concentrations from control plants (Nil) confirming lack of endophyte infection and displaying variability of Si in foliar tissue.

Whenever endophyte effects on foliar Si concentration were observed, positive relationships between *Epichloë* mycelial mass and foliar Si concentrations were also detected in both tall fescue (i.e., AR584 and WT strains; Figure 3A) and perennial ryegrass (i.e., AR37 strain; Figure 3B).

**FIGURE 4:**
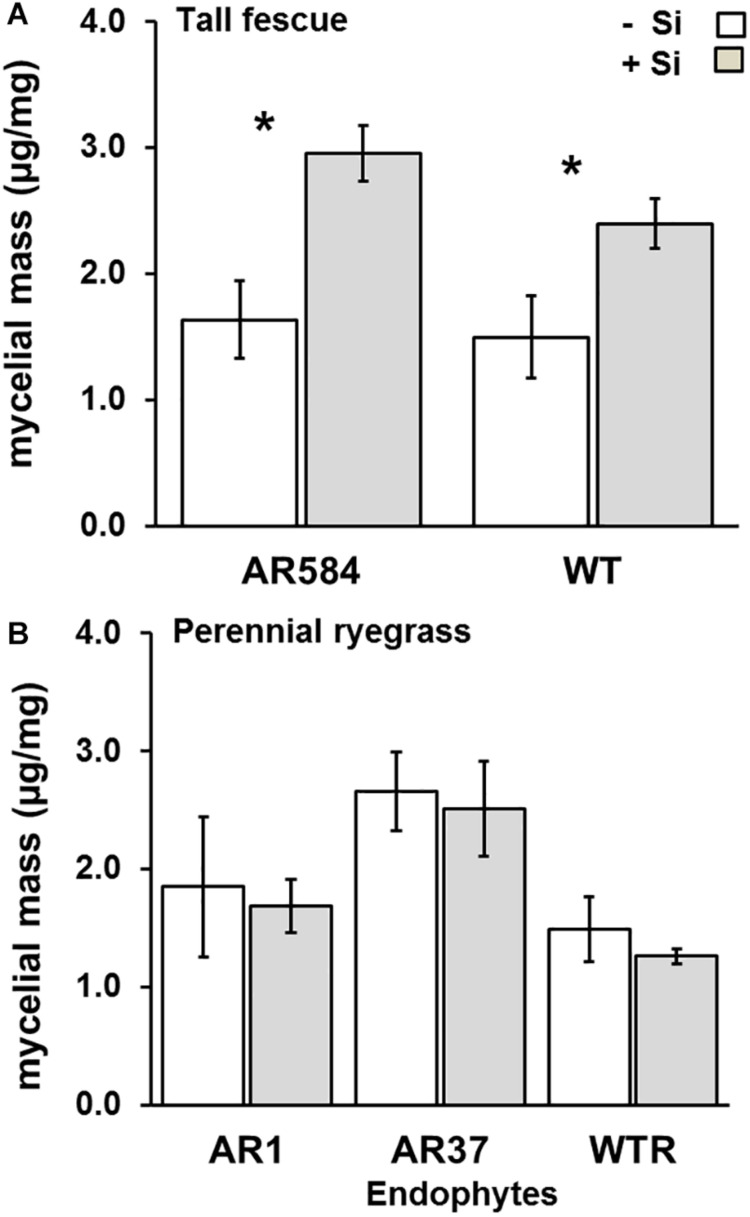
Variation in *Epichloë* endophyte colonization for **(A)** tall fescue and **(B)** perennial ryegrass with colonized different *Epichloë* endophyte strains growing hydroponically in the absence (–Si) or presence (+Si) of silicon (Si). Mean values ± standard error shown with N indicated within each bar. N is unbalanced due insufficient material for ELISA (see %Supplementary Table S2). Asterisks indicate significant differences (*P* < 0.05) within each endophyte strains.

Lastly, there was a significant effect of Si supply on endophyte colonization in tall fescue, but not for perennial ryegrass. Specifically, tall fescue plants in the +Si treatment had 80 and 60% higher mycelial masses than their Nil counterparts for AR584 (*F*_1_,_13_ = 11.52, *P* = 0.004) and WT (*F*_1_,_10_ = 5.54, *P* = 0.05) strains, respectively (Figure 4A). For perennial ryegrass, the effects of Si on endophyte mycelial mass were non-significant for all endophyte strains tested (AR1: *F*_1_,_14_ = 0.06, *P* = 0.80, AR37: *F*_1_,_15_ = 0.08, *P* = 0.77, WT: *F*_1_,_13_ = 0.32, *P* = 0.56) (Figure 4B).

## Discussion

In this study, we provide evidence that some *Epichloë* endophyte strains increased Si concentrations in the foliage of the two most common pasture grasses. Further we show that, whenever endophyte effects on foliar Si concentration were observed, the magnitude of this increase is positively associated with the amount of *Epichloë* mycelia in the host plant, and is independent of endophyte stimulation of plant growth. Moreover, using a hydroponic approach, which allows total Si exclusion (all soils contain at least some silicon), we provide novel evidence that Si-supply increases endophyte colonization in tall fescue.

While interactions varied depending on species and endophytic strain in perennial rygrass, reflecting some diversity and complexity in the relationship, for tall fescue, these results provide clear evidence of a reciprocally beneficial interaction between *Epichloë*-endophytes and Si. The novelty of these findings means that we have limited information about the possible mechanisms. Nonetheless, relevant studies suggest several mechanistic possibilities which we summarize below. While speculative, this provides a basis for future hypothesis-driven experiments including those conducted under more realistic field conditions.

### Foliar Si Concentration Increased With Some Endophyte Strains

Endophyte presence in tall fescue, and particularly the AR37 strain in perennial ryegrass, increased Si concentrations in foliar tissue of their host by more than 30%. This supports the only existing evidence for such effects, based on field data, by Huitu et al. (2014). Huitu et al. (2014) reported higher foliar Si concentrations in endophyte-colonized meadow fescue (*Schedonorus pratensis*) field plots compared to endophyte-free plots. While providing original evidence of a relationship between *Epichloë* and Si, Huitu et al. (2014) could not determine whether increases in foliar Si arose indirectly through endophyte induced changes in plant traits (e.g., increased root mass). Moreover, as a field soil-based study, Si supply could not be controlled, so it was unclear whether Si affected endophyte colonization. Accurate quantification of plant growth and full control of Si-supply, using the current hydroponic approach, allowed these questions to be addressed in the present study.

In this study, *Epichloë*-mediated increases in Si were independent of responses in root growth and positively correlated with endophyte colonization, supporting evidence for an intrinsic endophyte effect. One possibility is that symbionts may increase plant transpiration rates resulting in higher passive uptake of Si (Frew et al., 2017; Dastogeer, 2018). For instance, arbuscular mycorrhizae (AM) fungi (Frew et al., 2017), nitrogen fixing bacteria (Hartley and Gange, 2009) and *Epichloë* endophytes (Llorens et al., 2019) have all been reported to increase plant growth and modify photosynthetic processes through increased stomatal conductance (Malinowski and Belesky, 2000). In line with this, symbionts might also directly affect number and activity of plant aquaporins (water channels that facilitate transport of water between cells) which could promote active Si uptake. For instance, expression of transporter genes in hosts associated with nickel accumulation can be altered by *Epichloë* (Mirzahossini et al., 2015). Similarly, observed increases in Si could possibly be related to symbionts having aquaporins that operate similarly to those taking-up Si in plant roots (Frew et al., 2017). Interestingly, when elemental composition of AM-fungi spores and extraradical mycelia was investigated, AM-fungi displayed selective uptake of various elements, including Si (Hammer et al., 2011). Unfortunately, to our knowledge, the elemental composition of *Epichloë*-structures has not been investigated so far.

Another putative mechanism could be through endophyte-mediated alteration of host endogenous defense, or defense-priming (Bastias et al., 2017). Essentially, to maintain functional symbiosis, symbionts increase endogenous levels of the defense hormone jasmonic acid (JA) in their host plant (Martinez-Medina et al., 2016). This induction has been reported for obligate symbionts including AM-fungi (Jung et al., 2012), rhizobial bacteria (Dean et al., 2014), as well as, *Epichloë* endophytes (Bastias et al., 2017). Triggering the JA-pathway, either using chemical stimulation (methyl-jasmonate) or authentic herbivory, has been shown to promote Si-uptake (Ye et al., 2013; Kim et al., 2014; Hall et al., 2019, 2020). Consequently, the resulting spike in JA mediated by *Epichloë* endophyte symbiosis establishment, may lead to enhanced Si-uptake. However, JA-mediated increases in Si, while significant, have been shown to be relatively small, e.g., 12 and 10% as reported in Hall et al. (2020, 2019, respectively), compared to > 30% increased Si concentration found in this study. This suggests that the stimulation of JA pathway alone is unlikely to be the sole mechanism for increased foliar Si observed in endophyte-symbiotic plants.

Moreover, morphological changes in hosts as a result of endophyte infection have resulted in, for instance, greater number of vascular bundles in perennial ryegrass (Franco et al., 2020). In line with this, Si has been reported in high concentrations both in the vascular bundles of perennial ryegrass (Dinsdale et al., 1979) and in tall fescue (Vandegeer et al., 2020). Thus, indirect morphological changes in hosts as a result of endophyte infection might further increase Si in host tissues. Further, the amount of Si in grass leaves is known to differ greatly between grass species (Massey et al., 2007), including fescue (Buckner et al., 1967) and perennial ryegrass (Buckner et al., 1967; Moore, 1984). And particularly for tall fescue the amount of Si in grass leaves also varies between varieties, with harsh varieties having higher Si content and more spines on their leaf surface than soft varieties (Hartley et al., 2015). However, for fescue and ryegrass comparable amount of Si concentrations were found for *Festuca ovina* and *Lolium perenne* in both controlled (Massey and Hartley, 2009) and field conditions (Massey and Hartley, 2006), and has been directly linked with increasing leaf abrasiveness, thus reduced herbivory (Massey and Hartley, 2006; Massey et al., 2006, 2007). Thus, morphological changes in hosts by both endophyte and Si deposition might be strain and variety specific, highlighting the complexity of these interactions even further.

### Si Supply Increases Endophyte Colonization in Tall Fescue

Si supply did not reduce endophyte colonization in tall fescue nor in perennial ryegrass. This suggests that endophyte development in the apoplast of leaves is not hindered by Si deposition. Instead, endophyte colonization in tall fescue was actually increased by Si supply. The reasons for this remain speculative, but the natural evolution of a positive/neutral interaction is not unlikely, since grasses have a long evolutionary history with both endophytes (Young et al., 2014) and Si (Cooke and Leishman, 2012).

Si incorporation into cell walls is known to alter its structure (Kumar et al., 2017), and negative correlations between Si, cellulose and lignin have been reported (Klotzbücher et al., 2018). Likewise, *Epichloë* endophytes have been shown to decrease concentrations of lignin in perennial ryegrass regardless of plant genotype, and in contrast, showed no effect for tall fescue (Soto-Barajas et al., 2016). *Epichloë*-effects on host lignin are related to intracellular hyphae obtaining a supplementary source of carbon through hydrolysis of cell wall components (carbohydrates) (Rasmussen et al., 2012). Thus, if Si-supplemented plants have reduced lignin, this might affect perennial ryegrass endophyte more so than tall fescue. Thus, instead of interfering with endophyte colonization, Si deposition might in fact create additional niches for *Epichloë* mycelium (Christensen et al., 2008).

Further, Si accumulation in cell walls *in lieu* of carbon-rich structural constituents may also make more carbon available for metabolic processes (Cooke and Leishman, 2011), which could be another mechanism by which Si supply increased endophyte colonization in tall fescue. Likewise, since the endophytic fungal partner benefits from the symbiosis withdrawing assimilated photosynthetic carbon, the greater availability of carbon might strengthen the mutualism between *Epichloë* and its host grass (Rozpa̧dek et al., 2015). Lastly, higher mycelial mass was correlated with increased production of loline-protective alkaloids in *Schedonorus pratensis* (Cagnano et al., 2020). It is therefore also possible that Si-supply might increase endophyte produced alkaloids in tall fescue, at least, but this remains to be investigated.

## Conclusion

There is increasing dependency on grass monocultures (e.g., tall fescue) due to reduced natural grasslands suitable for forage; therefore sustainable strategies such as use of novel endophytes or Si supplementation to provide better tolerance to biotic stressors are required. Our results provide novel evidence that some *Epichloë* grass associations increase foliar Si concentrations in both tall fescue and perennial ryegrass, that Si supply can increase *Epichloë* colonization, and that these effects depend on the specific host species–endophyte strain association considered.

These reciprocal benefits of *Epichloë* endophytes and Si accumulation in tall fescue were observed in both WT (mammalian-toxic) and, more importantly, for the animal-safe (novel) AR584 *Epichloë* strains. Naturally, this needs to occur without major effects on digestibility for livestock, which needs to be further explored. While Si has been reported to cause small reductions in digestible organic matter (Van Soest and Jones, 1968; O’Reagain and Mentis, 1989), it’s considered that many ruminants are able to cope with increased Si in forage crops (Jones and Handreck, 1965; Melzer et al., 2010; Vandevenne et al., 2013). Consequently, the potential for greater resilience to stress by combining endophyte and Si is clear, particularly for tall fescue.

Moreover, tall fescue-endophyte associations seem to increase resistance to stresses more so than those perennial ryegrass–endophyte associations (Young et al., 2013). Both endophytes and Si accumulation can individually suppress pests but the current findings, albeit from highly controlled conditions, suggest there is at least the potential for them to work in combination. Future studies should explore these interactions with the inclusion of herbivores and in natural field conditions to determine whether this potential can be realized.

## Data Availability Statement

The original contributions presented in the study are included in the article/Supplementary Material, further inquiries can be directed to the corresponding author.

## Author Contributions

XC-S, JP, and SJ planned and designed the research. XC-S conducted the experimental work, collected the data, and analyzed the data with input from JP and SJ. XC-S led the writing of the manuscript with significant input from SJ, JP, FL, AP, and SH. All authors contributed to the article and approved the submitted version.

## Conflict of Interest

The authors declare that the research was conducted in the absence of any commercial or financial relationships that could be construed as a potential conflict of interest.
