# Supplementary material for: Reciprocal Effects of Silicon Supply and Endophytes on Silicon Accumulation and Epichloë Colonization in Grasses
Source: Front Plant Sci. 2020 Oct 27;11:593198. doi: 10.3389/fpls.2020.593198 (PMC7652995; doi:10.3389/fpls.2020.593198)
Supplement: Supplementary file 1 [file Data_Sheet_1.docx]

Supplementary Material

# Supplementary Data

Provided as an CVS file.

# Supplementary Tables

**Table S1**. Treatment combinations; Tall fescue (*Festuca arundinacea*) or perennial ryegrass (*Lolium perenne*) varieties used in the experiments with their corresponding *Epichloë*-strain growing hydroponically with (+Si) or without silicon (-Si). Initial and final replication (after removal of inappropriately attributed endophyte status).

| **Treatment combinations** | **Grass species - variety** | **Endophyte** | **Si** | **Initial replication** | **Final replication** |
| --- | --- | --- | --- | --- | --- |
| 1 | **Tall fescue- Fortuna** | **Nil** | **Si+** | 23 | 23* |
| 2 |  |  | **Si-** | 20 | 20* |
| 3 |  | **AR584** | **Si+** | 20 | 19* |
| 4 |  |  | **Si-** | 20 | 20* |
| 5 |  | **WT** | **Si+** | 10 | 6 |
| 6 |  |  | **Si-** | 10 | 8 |
| 7 | **Perennial ryegrass- Sumson** | **Nil** | **Si+** | 10 | 10 |
| 8 |  |  | **Si-** | 10 | 8 |
| 9 |  | **AR1** | **Si+** | 10 | 8 |
| 10 |  |  | **Si-** | 10 | 8 |
| 11 |  | **AR37** | **Si+** | 10 | 9 |
| 12 |  |  | **Si-** | 10 | 8 |
| 13 |  | **WT** | **Si+** | 10 | 9 |
| 14 |  |  | **Si-** | 10 | 10 |

*Conducted in two stages, separated by 4 weeks. The first stage was limited to a subset of tall fescue genotypes: ten or thirteen replicates of either non symbiotic (Nil) or symbiotic-AR584 tall fescue cv. Fortuna in a factorial combination with and without Si; resulting in 4 treatment combinations. The second stage included ten replicates of all *Epichloë* by genotype combinations tested in a factorial combination with and without Si. Stages were accounted for in models; interactions between RUN * SI for a) log silicon concentrations (F_1,77_= 0.008, *P*= 0.92), and b) shoot (F_1,77_= 0.006, *P*= 0.93) and c) root (F_1,77_= 1.180, *P*= 0.28) dry masses were not significant (two-way ANOVA).

**Table S2**. Tall fescue (*Festuca arundinacea*) or perennial ryegrass (*Lolium perenne*) varieties used in the experiments with their corresponding *Epichloë*-strain growing hydroponically with (+Si) or without silicon (-Si). Final replication for the sandwich ELISA, used as a measure of tissue-colonization is shown.

| **Treatment combinations** | **Grass species -variety** | **Endophyte** | **Si** | **Final replication** |
| --- | --- | --- | --- | --- |
| 1 | **Tall fescue- Fortuna** | **Nil** | **Si+** | 13 |
| 2 |  |  | **Si-** | 10 |
| 3 |  | **AR584** | **Si+** | 7 |
| 4 |  |  | **Si-** | 8 |
| 5 |  | **WT** | **Si+** | 5 |
| 6 |  |  | **Si-** | 5 |
| 7 | **Perennial ryegrass-Sumson** | **Nil** | **Si+** | 10 |
| 8 |  |  | **Si-** | 8 |
| 9 |  | **AR1** | **Si+** | 8 |
| 10 |  |  | **Si-** | 8 |
| 11 |  | **AR37** | **Si+** | 9 |
| 12 |  |  | **Si-** | 8 |
| 13 |  | **WT** | **Si+** | 5 |
| 14 |  |  | **Si-** | 10 |


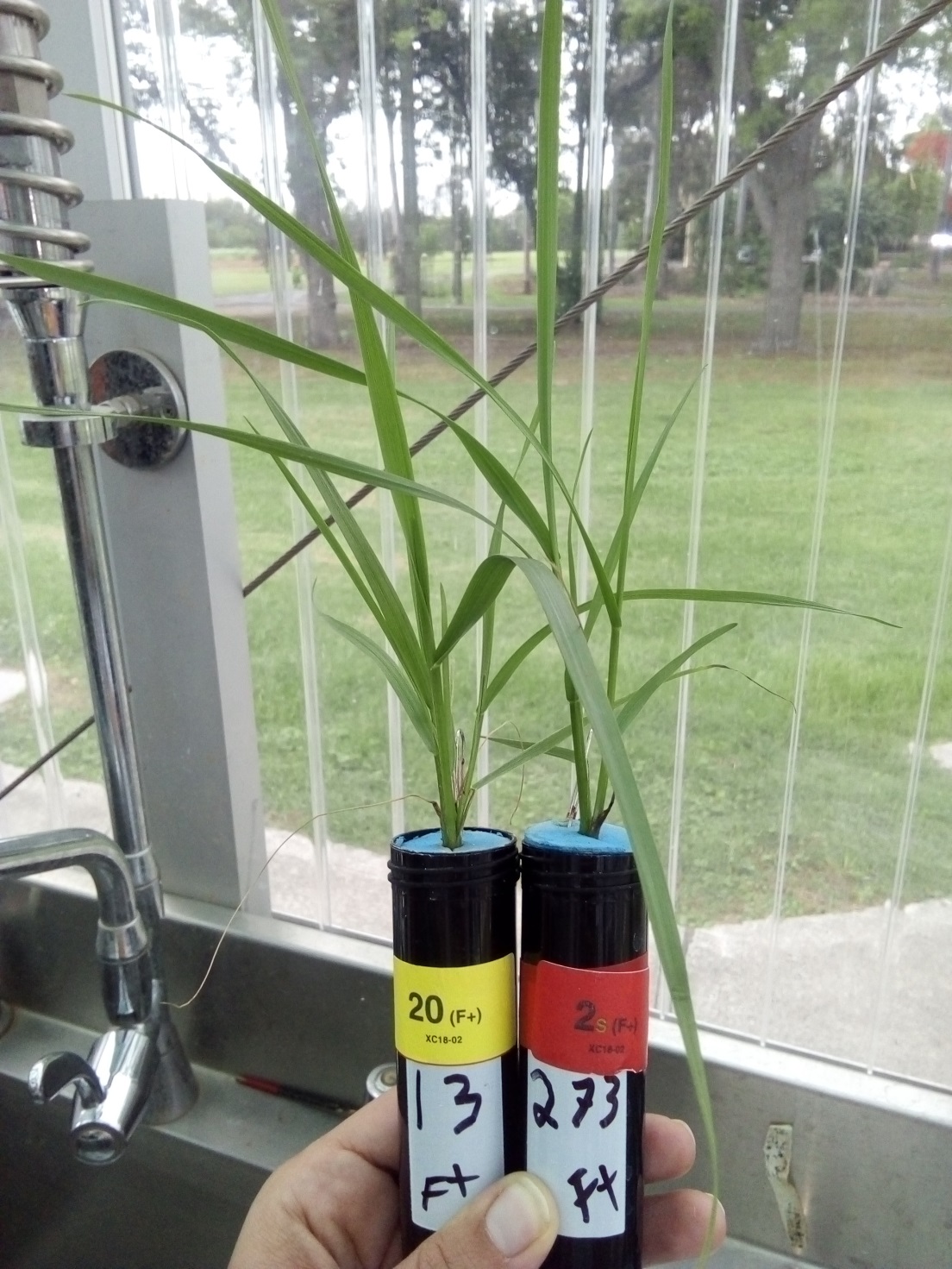


Figure S1. Example of the status of the plants immediately before harvest.
